# The effects of ginger supplementation on common gastrointestinal symptoms in patients with relapsing-remitting multiple sclerosis: a double-blind randomized placebo-controlled trial

**DOI:** 10.1186/s12906-023-04227-x

**Published:** 2023-10-27

**Authors:** Sahar Foshati, Maryam Poursadeghfard, Zahra Heidari, Reza Amani

**Affiliations:** 1 Nutrition Research Center, Department of Clinical Nutrition, School of Nutrition and Food Sciences, Shiraz University of Medical Sciences, Shiraz, Iran; 2 Clinical Neurology Research Center, Shiraz University of Medical Sciences, Shiraz, Iran; 3 Department of Biostatistics and Epidemiology, School of Health, Isfahan University of Medical Sciences, Isfahan, Iran; 4 Department of Clinical Nutrition, School of Nutrition and Food Science, Isfahan University of Medical Sciences, Isfahan, Iran

**Keywords:** Abdominal pain, Bloating, Constipation, Ginger, Multiple sclerosis, Nausea

## Abstract

**Background:**

Gastrointestinal (GI) symptoms affect more than 80% of individuals with relapsing-remitting multiple sclerosis (RRMS). Ginger is widely known for its GI relieving properties. Therefore, we investigated the effect of ginger supplementation on common GI symptoms in RRMS patients.

**Methods:**

This study was a 12-week double-blind parallel randomized controlled trial with a 3-week run-in period. The intervention (n = 26) and control (n = 26) groups received 500 mg ginger and placebo (as corn) supplements 3 times a day along with main meals, respectively. At the beginning and end of the trial, the frequency and severity of constipation, dysphagia, abdominal pain, diarrhea, bloating, belching, flatulence, heartburn, anorexia, and nausea were assessed using the visual analogue scale ranging from 0 to 100 mm. Totally, 49 participants completed the study. However, data analysis was performed on all 52 participants based on the intention-to-treat principle.

**Results:**

In comparison with placebo, ginger supplementation resulted in significant or near-significant reductions in the frequency (-23.63 ± 5.36 vs. 14.81 ± 2.78, *P* < 0.001) and severity (-24.15 ± 5.10 vs. 11.39 ± 3.23, *P* < 0.001) of constipation, the frequency (-12.41 ± 3.75 vs. 3.75 ± 1.82, *P* < 0.001) and severity (-13.43 ± 4.91 vs. 6.88 ± 2.69, *P* = 0.001) of nausea, the frequency (-9.31 ± 4.44 vs. 1.56 ± 4.05, *P* = 0.098) and severity (-11.57 ± 5.09 vs. 3.97 ± 3.99, *P* = 0.047) of bloating, and the severity of abdominal pain (-5.69 ± 3.66 vs. 3.43 ± 3.26, *P* = 0.069).

**Conclusion:**

Ginger consumption can improve constipation, nausea, bloating, and abdominal pain in patients with RRMS.

**Trial Registration:**

This trial was prospectively registered at the Iranian Registry of Clinical Trials (www.irct.ir) under the registration number IRCT20180818040827N3 on 06/10/2021.

## Background

Multiple sclerosis (MS) is a chronic neurodegenerative disease of autoimmune origin [1]. This disease has four clinical course patterns, with relapsing-remitting MS (RRMS) being the most prevalent [2]. MS typically leads to persistent disability and low quality of life due to disrupted communication between the brain and the body [1]. Added to this burden, recent evidence has shown that gastrointestinal (GI) symptoms such as abdominal pain, dysphagia, constipation, diarrhea, bloating, belching, flatulence, heartburn, anorexia, and nausea are common among patients with MS [3]. It seems that GI symptoms affect more than 80% of MS patients and are caused by disease complications, oral disease-modifying therapies, or both [4, 5]. In particular, anorectal dysfunction and slow colonic transit leading to constipation appear to be the most prevalent GI problems in individuals with MS [4]. After spasticity and incoordination, bowel dysfunction is rated by MS patients as the third most important factor limiting their ability to work [6].

Ginger (*Zingiber officinale*) is a herbaceous perennial plant belonging to the Zingiberaceae family [7]. Its edible rhizome (horizontal underground stem) is widely used as an aromatic spice and vegetable in both fresh and dried forms around the world [8]. Ginger rhizome or simply ginger is also a nutraceutical known for its antioxidant, neuroprotective, anti-diabetic, anti-inflammatory, immunomodulatory, anti-obesity, cardioprotective, anti-cancer, analgesic, and GI protective properties [9–12]. In particular, supplementation with ginger has shown beneficial effects on nausea, vomiting, dyspepsia, bloating, gastroenteritis, GI malignancies, abdominal discomfort, gastric dysrhythmias, gastric ulcers, gastric emptying, intestinal motility, and swallowing function [12, 13]. For instance, ginger consumption can relieve most symptoms of functional dyspepsia such as early satiety, fullness, belching, nausea, epigastric pain, and heartburn [14]. Also, ginger consumption has been reported to accelerate GI motility and reduce food transit time, which may alleviate constipation [15, 16]. In addition, salivary concentrations of substance P and swallowing function scores have improved after ginger supplementation [17].

Considering the high prevalence of GI problems in MS patients and the positive effects of ginger on the digestive system [4, 12], we sought to design a clinical trial and investigate the effect of ginger supplementation on the frequency and severity of common GI symptoms in RRMS patients.

## Materials and methods

### Trial design

This study was a 12-week double-blind parallel randomized placebo-controlled trial with a 3-week run-in period. It was conducted in agreement with the Declaration of Helsinki and its later amendments. Eligible patients were recruited from Fars MS Association and Imam Reza (A.S) Clinic located in Shiraz, Iran. Before the enrollment, informed consent was obtained from the participants. This trial was approved by the Medical Ethics Committee at the Isfahan University of Medical Sciences under the ethics code IR.MUI.RESEARCH.REC.1400.248. Also, it was prospectively registered at the Iranian Registry of Clinical Trials (www.irct.ir) under the registration number IRCT20180818040827N3 on 06/10/2021.

### Participants

Based on the sample size calculation described in detail in our published study protocol [18], 52 people (26 in each intervention and control group) were recruited. The inclusion criteria were subjects diagnosed with RRMS according to the latest revision of the McDonald criteria [19], men or non-menopausal women aged 18 to 50 years old, a score of ≤ 4.5 in the Expanded Disability Status Scale [20], no MS relapse or corticosteroid therapy for the past 3 months, no changes in the type or dose of MS medications for the past 6 months, and the willingness and ability to participate in this study. The exclusion criteria were patients with other autoimmune disorders or diagnosed cancers, pregnancy, MS relapse or corticosteroid therapy during the trial, changes in the type or dose of MS medications throughout the trial, allergic reactions to ginger or placebo (corn), supplementation with antioxidants or nutrients (except vitamin D), and consumption of < 90% of ginger or placebo tablets.

### Randomization and blinding

Eligible RRMS patients were assigned to ginger and placebo groups using stratified permuted block randomization (allocation ratio = 1:1, block size = 4). The stratification was performed according to gender. The following website was used for the generation of random allocation sequence: https://www.sealedenvelope.com/simple-randomiser/v1/lists. Tablets of ginger and placebo were packed in sequentially numbered identical bottles according to the allocation sequence. The sequence generation, allocation concealment, and assignment of subjects to the groups were implemented by different trained individuals. The researchers, participants, care providers, and outcome assessors were blinded to group assignment until trial completion. It is noteworthy that ginger and placebo tablets were exactly the same in package, size, shape, and color. Moreover, a very small amount of ginger powder was added to bottles containing placebo tablets to give ginger odor, as previously done by Mahluji et al. [21] and Ebrahimzadeh Attari et al. [22].

### Run-in period

Before random allocation, eligible participants underwent a three-week run-in period. During this time, RRMS patients were requested to avoid consuming ginger and its products and to maintain their usual physical activity and dietary intake.

### Intervention

The treatment and control groups received 500 mg ginger and placebo (corn) tablets thrice a day with main meals for 12 weeks, respectively. Each 500 mg ginger tablet was standardized to contain 25 mg gingerols. Ginger and placebo supplements were provided by Dineh Iran Industries Complex, Tehran, Iran. During the trial, both groups were asked to maintain their usual diet and physical activity and to avoid consuming ginger and other products containing it. In addition, possible side effects of the intervention were evaluated according to the trial protocol [18].

### GI symptoms

At the beginning and end of this trial, the frequency and severity of constipation, dysphagia, abdominal pain, diarrhea, bloating, belching, flatulence, heartburn, anorexia, and nausea were assessed using the visual analogue scale (VAS) ranging from 0 mm (none) to 100 mm (worst possible). This assessment tool has previously been applied and validated by Preziosi et al. to evaluate GI symptoms in patients with MS [23].

### Physical activity and dietary intake

Physical activity and dietary intake can affect GI symptoms [24]. Thus, we assessed them at the beginning and end of this study. Physical activity levels per week were evaluated using the International Physical Activity Questionnaire, which has acceptable reliability and validity in Iranians [25]. Dietary intakes were collected using three-day food records (two weekdays and one weekend day). Then, energy and nutrient intake per day were determined by Nutritionist IV software customized for Iranian foods (version 3.5.2, The Hearst Corporation, San Bruno, California, United States).

### Statistical analysis

Data analysis was performed according to the intention-to-treat (ITT) principle using IBM SPSS Statistics software (version 26, IBM Corporation, Armonk, New York, United States). The expectation-maximization algorithm was run to impute missing values in ITT analysis [26]. Number (percentage) was used to report qualitative variables, and mean ± standard error was used to represent quantitative variables. The Shapiro-Wilk test and skewness and kurtosis statistics were used to check the normality of quantitative variables. Non-normally distributed data were transformed using suitable functions including logarithm and square root. Between-group comparisons of demographic and medical characteristics, dietary intakes, and physical activity were performed using the independent t-test, chi-square test, or Fisher’s exact test as appropriate. Within-group comparisons of GI symptoms were done using the paired t-test. Between-group comparisons of GI symptoms were done using the analysis of covariance adjusted for the baseline values. All analyses were two-tailed with a significance level of 0.05.

## Results

**Fig. 1:**
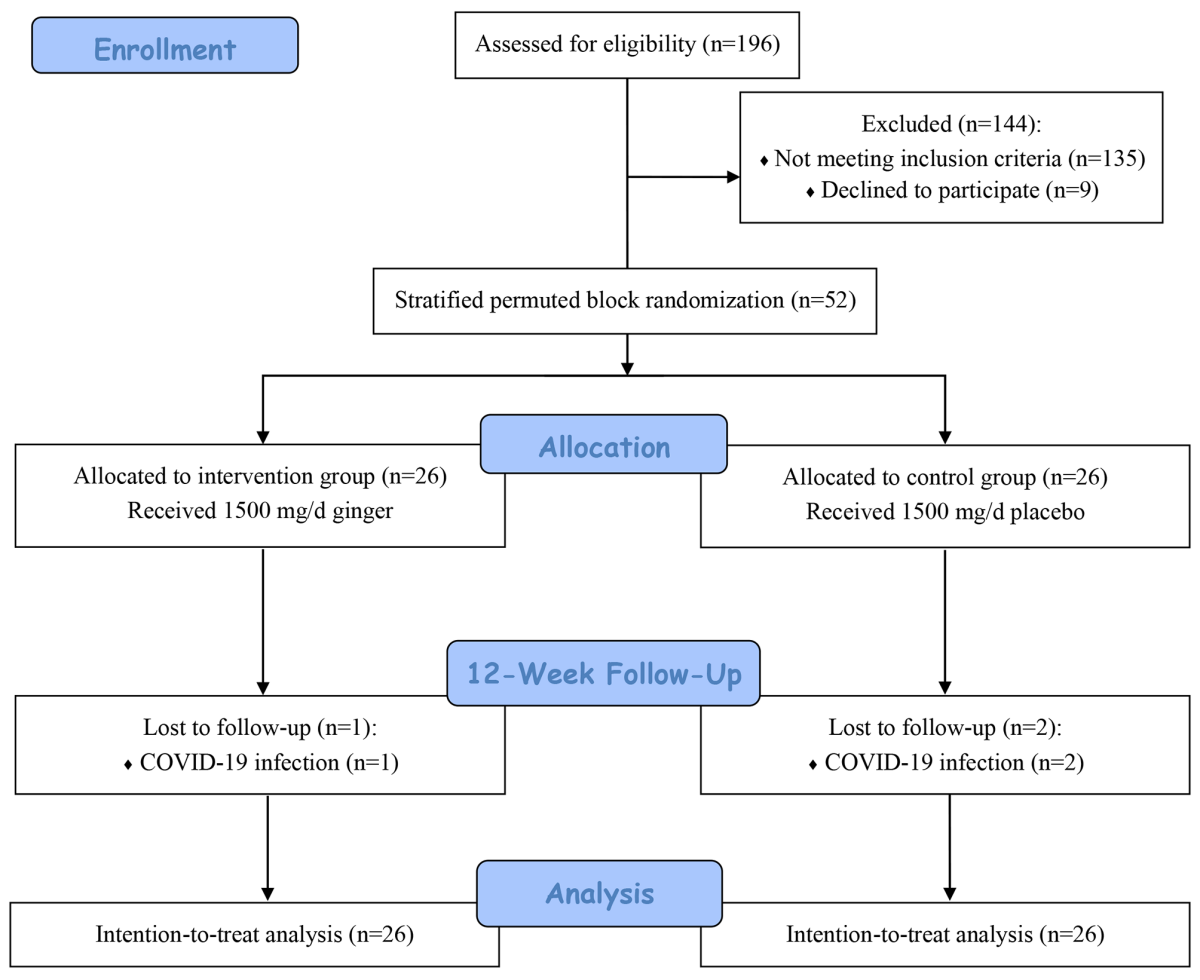
The flow diagram of the participants throughout the study

This clinical trial was conducted in 2022. From 196 RRMS patients assessed for eligibility, 52 met the inclusion criteria and participated in the study. The participants were randomly assigned to receive either placebo (n = 26) or ginger tablets (n = 26). During the trial, two patients in the control group and one patient in the intervention group dropped out due to COVID-19 infection, a systemic disease with GI symptoms [27]. However, all 52 participants were analyzed based on ITT principle (Fig. 1).

None of the participants were receiving GI medications. In addition, there was no significant difference between the intervention and control groups in terms of demographic and medical characteristics (Table 1). Moreover, energy and nutrient intakes as well as physical activity levels were not significantly different between the two groups (Table 2).

**Table 1:** Demographic and medical characteristics of the RRMS patients

	Ginger (n = 26)	Placebo (n = 26)	*P*-value ^a^
Age (year)	36.5 ± 1.2	35.0 ± 1.4	0.41
Female	20 (76.9)	20 (76.9)	> 0.99
Married	21 (80.8)	17 (65.4)	0.35
University education	12 (46.2)	16 (61.5)	0.40
Occupation			0.41
Housewife	14 (53.8)	8 (30.8)	
Employee	4 (15.4)	5 (19.2)	
Freelance	5 (19.2)	9 (34.6)	
Unemployed	3 (11.5)	4 (15.4)	
Smoker	5 (19.2)	6 (23.1)	> 0.99
Alcohol drinker	4 (15.4)	5 (19.2)	> 0.99
Received vitamin D supplement	21 (80.8)	22 (84.6)	> 0.99
Received antidepressants or sedatives	9 (34.6)	9 (34.6)	> 0.99
BMI categories			0.84
Underweight	3 (11.5)	1 (3.8)	
Normal weight	9 (34.6)	11 (42.3)	
Overweight	10 (38.5)	10 (38.5)	
Obesity	4 (15.4)	4 (15.4)	
Disease duration (year)	6.5 ± 0.9	6.4 ± 1.0	0.98
Disease-modifying therapies			0.48
Oral	7 (26.9)	11 (42.3)	
Injection	7 (26.9)	7 (26.9)	
Infusion	11 (42.3)	6 (23.1)	
None	1 (3.8)	2 (7.7)	

Note: Data are expressed as mean ± standard error or number (percentage)

Abbreviations: BMI, body mass index; RRMS, relapsing-remitting multiple sclerosis

^a^ Obtained from the independent t-test for quantitative variables and the chi-square test or Fisher’s exact test for qualitative variables

**Table 2:** Dietary intake and physical activity of the subjects throughout the trial

	Ginger (n = 26)	Placebo (n = 26)	*P*-value ^a^
Energy (kcal/d)	2194 ± 98	2276 ± 136	0.63
Carbohydrate (g/d)	304.2 ± 16.1	310.7 ± 19.6	0.80
Total fat (g/d)	76.86 ± 4.16	82.14 ± 6.78	0.78
Protein (g/d)	76.23 ± 3.79	81.00 ± 5.92	0.68
Dietary fiber (g/d)	16.94 ± 1.15	18.63 ± 1.95	0.46
Lactose (g/d)	4.96 ± 1.26	4.21 ± 1.02	0.65
Fructose (g/d)	18.69 ± 2.02	20.46 ± 2.40	0.58
Glucose (g/d)	14.61 ± 1.90	14.27 ± 1.82	0.90
Vitamin C (mg/d)	114.0 ± 17.1	101.9 ± 17.4	0.62
Vitamin B1 (mg/d)	2.05 ± 0.13	1.89 ± 0.13	0.38
Vitamin B2 (mg/d)	1.74 ± 0.11	1.80 ± 0.15	0.95
Vitamin B3 (mg/d)	25.24 ± 1.36	25.38 ± 2.15	0.82
Vitamin B5 (mg/d)	5.09 ± 0.28	4.95 ± 0.33	0.74
Vitamin B6 (mg/d)	1.49 ± 0.08	1.51 ± 0.14	0.64
Vitamin B9 (µg/d)	270.1 ± 21.1	315.8 ± 37.1	0.52
Vitamin B12 (µg/d)	2.44 ± 0.27	2.71 ± 0.43	0.98
Vitamin A (RE/d)	452.8 ± 63.2	488.2 ± 76.2	0.76
Calcium (mg/d)	726.5 ± 57.3	622.4 ± 48.4	0.17
Magnesium (mg/d)	257.7 ± 13.6	285.9 ± 27.8	0.86
Sodium (mg/d)	1724 ± 138	1801 ± 156	0.71
Potassium (mg/d)	2420 ± 151	2608 ± 263	0.85
Phosphorus (mg/d)	1127 ± 57	1162 ± 65	0.69
Iron (mg/d)	15.63 ± 0.91	16.11 ± 1.07	0.74
Zinc (mg/d)	8.83 ± 0.46	9.21 ± 0.66	0.64
Copper (mg/d)	1.36 ± 0.09	1.42 ± 0.13	0.70
Selenium (mcg/d)	87 ± 11	70 ± 9	0.25
IPAQ (MET-min/wk)	963 ± 140	1013 ± 145	0.80

Note: Data are expressed as mean ± standard error

Abbreviations: IPAQ, International Physical Activity Questionnaire

^a^ Obtained from the independent t-test

The frequency of nausea (*P* = 0.003), bloating (*P* = 0.046), and constipation (*P* < 0.001) were significantly reduced after ginger supplementation compared with the baseline. In contrast, the frequency of nausea (*P* = 0.043), belching (*P* = 0.018), and constipation (*P* < 0.001) were significantly higher at the end of the study compared with the baseline in the control group. Also, the frequency of abdominal pain was marginally significantly (*P* = 0.078) greater at the 12th week in the control group. However, within-group differences in the frequency of other GI symptoms were non-significant (Table 3).

**Table 3:** Within- and between-group comparisons in the frequency of gastrointestinal symptoms during the 12-week intervention

	Ginger (n = 26)				Placebo (n = 26)				*P*-value ^b^
	Baseline	Endpoint	Change	*P*-value ^a^	Baseline	Endpoint	Change	*P*-value ^a^	
Anorexia	19.23 ± 4.45	19.48 ± 3.96	0.25 ± 3.40	0.51	13.46 ± 4.21	15.87 ± 4.56	2.41 ± 3.08	0.52	0.70
Dysphagia	4.81 ± 1.97	3.92 ± 1.80	-0.89 ± 2.94	0.77	11.54 ± 4.44	10.73 ± 3.95	-0.81 ± 1.69	0.64	0.44
Heartburn	22.11 ± 5.25	16.48 ± 5.69	-5.63 ± 4.46	0.22	11.54 ± 3.17	16.62 ± 4.55	5.08 ± 3.10	0.11	0.12
Nausea	13.46 ± 3.98	1.05 ± 0.96	-12.41 ± 3.75	0.003	5.77 ± 2.52	9.52 ± 3.08	3.75 ± 1.82	0.043	< 0.001
Abdominal pain	13.46 ± 3.98	11.70 ± 3.44	-1.76 ± 3.65	0.75	10.58 ± 4.20	14.75 ± 4.80	4.17 ± 2.26	0.078	0.25
Bloating	33.65 ± 5.54	24.34 ± 5.04	-9.31 ± 4.44	0.046	28.85 ± 5.31	30.41 ± 5.26	1.56 ± 4.05	0.70	0.098
Belching	21.15 ± 4.53	20.52 ± 4.11	-0.63 ± 3.81	0.87	13.46 ± 4.43	21.82 ± 5.41	8.36 ± 3.30	0.018	0.15
Flatulence	31.73 ± 4.71	31.38 ± 4.54	-0.35 ± 5.83	0.95	28.85 ± 4.74	28.15 ± 4.37	-0.70 ± 2.91	0.81	0.74
Constipation	39.42 ± 6.53	15.79 ± 4.98	-23.63 ± 5.36	< 0.001	18.27 ± 4.91	33.08 ± 5.77	14.81 ± 2.78	< 0.001	< 0.001
Diarrhea	6.73 ± 2.61	6.95 ± 2.20	0.22 ± 2.41	0.93	10.58 ± 3.71	11.16 ± 4.16	0.58 ± 4.74	0.90	0.53

Note: Data are expressed as mean ± standard error

Note: Data were measured using the visual analogue scale ranging from 0 to 100 mm

^a^ Obtained from the paired t-test

^b^ Obtained from the analysis of covariance adjusted for baseline values

In the intervention group, the severity of heartburn (*P* = 0.016), nausea (*P* = 0.005), bloating (*P* = 0.032), and constipation (*P* < 0.001) were significantly lower at the end of the study compared with the beginning. In the placebo group, the severity of nausea (*P* = 0.004) and constipation (*P* = 0.002) were significantly increased at the endpoint of the follow-up period compared with the baseline. However, within-group differences in the severity of other GI symptoms did not reach the significance level (Table 4).

**Table 4:** Within- and between-group comparisons in the severity of gastrointestinal symptoms during the 12-week intervention

	Ginger (n = 26)				Placebo (n = 26)				*P*-value ^b^
	Baseline	Endpoint	Change	*P*-value ^a^	Baseline	Endpoint	Change	*P*-value ^a^	
Anorexia	26.92 ± 7.19	22.61 ± 5.60	-4.31 ± 5.59	0.45	14.23 ± 4.42	19.65 ± 5.44	5.42 ± 4.25	0.21	0.48
Dysphagia	2.50 ± 1.08	2.54 ± 1.36	0.04 ± 1.88	0.90	11.54 ± 4.37	9.89 ± 4.28	-1.65 ± 2.68	0.77	0.44
Heartburn	28.65 ± 7.29	14.51 ± 5.46	-14.14 ± 5.45	0.016	16.35 ± 5.14	15.94 ± 5.05	-0.41 ± 5.06	0.88	0.12
Nausea	14.81 ± 5.00	1.38 ± 1.34	-13.43 ± 4.91	0.005	3.46 ± 1.81	10.34 ± 4.19	6.88 ± 2.69	0.004	0.001
Abdominal pain	12.89 ± 4.07	7.20 ± 2.40	-5.69 ± 3.66	0.13	10.19 ± 4.60	13.62 ± 5.17	3.43 ± 3.26	0.30	0.069
Bloating	36.35 ± 6.47	24.78 ± 5.40	-11.57 ± 5.09	0.032	26.35 ± 5.35	30.32 ± 5.86	3.97 ± 3.99	0.33	0.047
Belching	19.61 ± 5.06	19.83 ± 4.68	0.22 ± 2.43	0.93	16.73 ± 5.79	18.71 ± 4.98	1.98 ± 4.08	0.63	0.83
Flatulence	29.04 ± 4.88	31.07 ± 5.48	2.03 ± 6.60	0.76	25.96 ± 5.05	25.59 ± 3.86	-0.37 ± 3.44	0.92	0.49
Constipation	40.77 ± 7.41	16.62 ± 5.65	-24.15 ± 5.10	< 0.001	18.65 ± 5.47	30.04 ± 6.05	11.39 ± 3.23	0.002	< 0.001
Diarrhea	8.85 ± 4.32	5.76 ± 2.40	-3.09 ± 3.93	0.82	9.81 ± 3.75	10.67 ± 4.65	0.86 ± 4.60	0.87	0.57

Note: Data are expressed as mean ± standard error

Note: Data were measured using the visual analogue scale ranging from 0 to 100 mm

^a^ Obtained from the paired t-test

^b^ Obtained from the analysis of covariance adjusted for baseline values

As shown in Tables 3 and 4, supplementation with ginger caused significant reductions in the frequency (-12.41 ± 3.75 vs. 3.75 ± 1.82, *P* < 0.001) and severity (-13.43 ± 4.91 vs. 6.88 ± 2.69, *P* = 0.001) of nausea compared with placebo. In addition, the frequency (-23.63 ± 5.36 vs. 14.81 ± 2.78, *P* < 0.001) and severity (-24.15 ± 5.10 vs. 11.39 ± 3.23, *P* < 0.001) of constipation were significantly decreased in the intervention group compared with the control group. Also, a significant reduction in the severity of bloating (-11.57 ± 5.09 vs. 3.97 ± 3.99, *P* = 0.047) and a marginally significant reduction in the frequency of bloating (-9.31 ± 4.44 vs. 1.56 ± 4.05, *P* = 0.098) were observed in the ginger group compared to the placebo group. It is worth mentioning that constipation, nausea, and bloating were the most improved GI symptoms, respectively. Furthermore, the severity of abdominal pain was near-significantly lower following ginger supplementation in comparison with the controls (-5.69 ± 3.66 vs. 3.43 ± 3.26, *P* = 0.069). However, between-group differences in the frequency or severity of other GI symptoms were non-significant.

Minor side effects were seen during this clinical trial (7.7% in the ginger group vs. 11.5% in the placebo group). In the treatment group, one patient experienced abdominal discomfort, and another patient reported heartburn. In the control group, one patient reported headache, and two participants experienced heartburn.

## Discussion

The findings of this study indicated that 1500 mg/d ginger supplementation for 12 weeks statistically significantly reduced the frequency and severity of nausea and constipation as well as the severity of bloating. In addition, supplementation with ginger caused statistically near-significant reductions in the frequency of bloating and the severity of abdominal pain in patients with RRMS. Nevertheless, ginger consumption showed no significant impact on the frequency and severity of dysphagia, diarrhea, belching, flatulence, heartburn, and anorexia and the frequency of abdominal pain. It is noteworthy that the side effects of ginger in this study were rare and mild, representing a very good safety profile.

Interestingly, the highest reduction in GI symptoms after ginger supplementation belonged to constipation in this trial. Constipation is the most common GI problem in RRMS patients and is caused by prolonged colonic transit time and altered colonic compliance and basal tone [28]. These complications may be occurred due to demyelinating lesions of the conus medullaris or more proximal lesions [29]. After constipation, the highest reduction in GI symptoms following supplementation with ginger belonged to nausea and bloating, respectively. These two GI symptoms besides abdominal pain may be the result of indigestion (dyspepsia) in patients with RRMS. Evidence has shown that dyspepsia is about four times more prevalent in MS patients than the general population [3]. It is worth mentioning that the minimum clinically important difference, the smallest difference in an outcome that can clinically improve patient management, has been recommended to be 10 mm for VAS of GI symptoms [30]. Therefore, it appears that the improvement of constipation, nausea, and bloating in this trial was clinically significant as presented in Tables 3 and 4. Nevertheless, the improvement of the severity of abdominal pain seems to be not clinically of importance.

Consistent with our findings, previous studies have reported that ginger supplementation can improve constipation without causing diarrhea [31, 32]. For example, a recent trial showed that ginger supplementation significantly relieved constipation in patients with hypothyroidism [31]. In addition, another trial revealed that ginger consumption caused a significant reduction in time to first defecation after cesarean section in women [32]. The mechanism of this beneficial effect may be related to the ability of ginger to increase GI motility [15]. Among dietary spices, ginger is the most potent one to reduce colonic transit time. In more details, ginger seems to decrease food transit time by about 30% [16]. Also, a mechanistic study has indicated that ginger has both GI prokinetic and relaxant effects which are mediated through cholinergic agonism and calcium antagonism, respectively [33]. Furthermore, two ginger constituents, 6-gingerol and 6-shogaol, have transient receptor potential ankyrin 1 (TRPA1)-stimulating activity [34]. TRPA1 can promote serotonin release from enterochromaffin cells and subsequently increase colonic peristalsis and tone [35, 36]. Moreover, ginger has been reported to enhance circulating levels of gastrin, which its release seems to be impaired in patients with chronic constipation [37, 38]. In addition, ginger may raise circulating concentrations of ghrelin, which stimulates GI motility in mammals [39, 40].

The results of previous studies are controversial regarding the effect of supplementation with ginger on dyspepsia symptoms. Consistent with our findings, several trials reported positive effects of ginger on at least one dyspepsia symptom, such as nausea, bloating, and abdominal pain [14, 37, 41, 42]. Inconsistent with our findings, some trials showed no effect of ginger on any dyspepsia symptoms [43–45]. This contradiction may be due to differences in the duration of ginger consumption. Long-term studies (i.e., 2–4 weeks) found significant beneficial effects of ginger on dyspepsia symptoms [14, 37, 41, 42], but short-term studies (i.e., 1 day) did not [43–45]. Despite causing a significant reduction in food transit time, ginger supplementation has been shown to improve the digestion and absorption of macronutrients [46, 47]. This is because ginger can stimulate the activities of intestinal and pancreatic digestive enzymes including sucrase, maltase, amylase, lipase, acid phosphatase, trypsin, and chymotrypsin [48, 49]. Interestingly, this stimulatory effect has been observed only after long-term consumption of repeated doses of ginger, not after acute consumption of a single dose [49].

In line with our results, a systematic review of 109 clinical trials has indicated that ginger has a very good safety profile [7]. In addition, ginger supplementation at doses up to 4000 mg/d is generally recognized as safe (GRAS) [50]. Mild GI-related side effects such as mouth irritation, heartburn, abdominal discomfort, diarrhea, gas, and belching have rarely been reported after ginger consumption [7, 12]. Therefore, the GI benefits of ginger greatly outweigh its GI side effects.

In the authors’ opinion, the management of prevalent GI problems such as constipation and dyspepsia in MS patients can improve their quality of life, well-being, daily functioning, and work ability. The prokinetic effects of ginger can alleviate constipation, and the stimulatory effects of ginger on digestive enzymes can relieve dyspepsia in long-term use [33, 46, 47]. Also, the analgesic, carminative, anti-nausea, antioxidant, and anti-inflammatory properties of ginger may further increase its efficacy in reducing GI symptoms [12]. Therefore, we suggest ginger as a safe complementary therapy for MS patients with GI complications.

Finally, this trial has some strengths and limitations that should be acknowledged. The first strength is the novelty of the work. To the best of our knowledge, this study is the first one that investigated the effect of ginger supplementation on GI symptoms in patients with MS. The second strength is the assessment of key potential confounders including dietary intake and physical activity during the intervention. On the other hand, the first limitation is that this trial was conducted on RRMS patients; therefore, its findings may not be generalizable to other types of MS. The second limitation is that the frequency and severity of GI symptoms were mild to moderate in this study; therefore, it is unclear whether our results are applicable to more severe cases. These limitations need to be considered in the design of future clinical trials.

## Conclusion

In conclusion, ginger consumption seems to alleviate GI symptoms in patients suffering from RRMS. Particularly, ginger supplementation can improve constipation, nausea, bloating, and abdominal pain in patients with RRMS. Nevertheless, further clinical trials and mechanistic studies are necessary to confirm these findings.

## Data Availability

The data supporting the results of this trial are available from the corresponding author, upon reasonable request.

## List of Abbreviations

GI: Gastrointestinal
ITT: Intention-to-treat
MS: Multiple sclerosis
RRMS: Relapsing-remitting multiple sclerosis
TRPA1: Transient receptor potential ankyrin 1
VAS: Visual analogue scale
